# Life-history theory and climate change: resolving population and parental investment paradoxes

**DOI:** 10.1098/rsos.160470

**Published:** 2016-11-30

**Authors:** Mark Caudell, Robert Quinlan

**Affiliations:** 1 Paul G. Allen School for Global Animal Health, Washington State University, Pullman, WA 99164, USA; 2 Department of Anthropology, Washington State University, Pullman, WA 99164, USA

**Keywords:** life-history theory, climate change, ecological psychology, sustainability, environmental risk

## Abstract

Population growth in the next half-century is on pace to raise global carbon emissions by half. Carbon emissions are associated with fertility as a by-product of somatic and parental investment, which is predicted to involve time orientation/preference as a mediating psychological mechanism. Here, we draw upon life-history theory (LHT) to investigate associations between future orientation and fertility, and their impacts on carbon emissions. We argue ‘*K*-strategy’ life history (LH) in high-income countries has resulted in parental investment behaviours involving future orientation that, paradoxically, promote unsustainable carbon emissions, thereby lowering the Earth's *K* or carrying capacity. Increasing the rate of approach towards this capacity are ‘*r*-strategy’ LHs in low-income countries that promote population growth. We explore interactions between future orientation and development that might slow the rate of approach towards global *K*. Examination of 67 000 individuals across 75 countries suggests that future orientation interacts with the relationship between environmental risk and fertility and with development related parental investment, particularly investment in higher education, to slow population growth and mitigate *per capita* carbon emissions. Results emphasize that LHT will be an important tool in understanding the demographic and consumption patterns that drive anthropogenic climate change.

## 1. Introduction

Relationships between patterns of human fertility and anthropogenic climate change deserve more attention [1–6]. Population growth in the next half-century could raise global carbon emissions by half, even with the unlikely assumption of no *per capita* increases in emissions [7]. Among mammals, *per capita* energy consumption is generally negatively associated with fertility [8]. In humans, however, carbon emissions are positively associated with fertility rate increases *and* decreases depending on the development context [2,3]. We suggest that parental investment and life-history theory (LHT) may be relevant for understanding fertility effects on anthropogenic climate change. Parental investment in embodied capital that theoretically targets ‘dynastic fitness’ in industrial labour markets [9] results in large *per capita* energy investments [10] with potentially large corresponding carbon emissions that reduce global carrying capacity and long-term sustainability and survival probabilities. Early LHT distinguished between *r-* and *K*-strategies, relevant to density-dependent evolutionary dynamics (see [11]). *K* in our analysis refers to human ‘niche construction’ [12] effects (e.g. unsustainable energy use) that reduce global carrying capacity as an externality associated with high investments in embodied capital [9]. Similarly, *r* in our analysis characterizes nations with high fertility contributing to global population growth headed towards a shrinking *K*. We wonder if human life history (LH) perception can be tuned to anticipate impending global density-dependent shocks [13].

Current LHT predicts delay discounting in rapidly growing populations with high mortality [14–17]. Conversely, we expect that ‘future orientation’ is a phenotypic property of individuals in low mortality, low fertility, high parental investment environments, where planning and long-term goals influence differential fitness. Paradoxically, future orientation and associated LH regimes entail externalities, such as carbon emissions, that contribute to environmental uncertainty. Are there any conditions under which investment in embodied capital can proceed with fewer unintended costs? Using data from 67 000 women across 75 countries, we examine associations between future orientation and carbon emissions in different LH and development regimes (high income versus low income).

## 2. Background

### 2.1. Life history, risk and reproduction

In LHT, behaviour is guided by the costs and benefits of investing energy into ‘competing’ life functions of growth, development, maintenance, repair (i.e. somatic or embodied effort) and reproduction [18,19]. As resources invested into one life function cannot be devoted to another, a set of trade-offs arises [20]. The trade-off between current and future reproduction underlies *r*- and *K-*strategies [19,21]. *K-*strategies are typified by protracted periods of investment in somatic effort and delayed onset and pace of reproduction, which allow individuals to produce relatively few ‘high-quality’ offspring. In the context of high-income and industrialized countries, the pursuit of *K*-strategies results in large *per capita* emissions per offspring. By contrast, *r-*strategies include earlier and more frequent reproduction, and reduced per child investments [18,19,22,23], and so should be associated with smaller emissions *per capita*. In sum, our use of *r* and *K* captures some of the density-dependent dynamics of early models [21,24] and more generally refers to later LH paradigms [25,26]. Unlike *K-*selection as originally conceived for density-dependent regimes as populations reach equilibrium, which globally humanity does not yet exhibit [27], we assume focus on *K* is also relevant to human adaptation when equilibrium assumptions are relaxed. Generally, attention to non-equilibrium dynamics appears to be a promising avenue for human evolutionary and social ecology [28–30].

In part, local extrinsic risk structures LH [20,31,32]. Extrinsic risk refers to relatively less-predictable hazards (e.g. civil war, extreme weather, some epidemics), such that an organism is unable to alter survival probabilities through allocation of effort, both for themselves and their offspring. High extrinsic risk favours *r*-strategies to ‘beat the odds’ that a parent dies before reproduction or leaves relatively few offspring given high juvenile mortality. In low risk environments, differential reproductive success is more contingent upon somatic (embodied) investment, thereby favouring *K*-strategies [18,19,32]. Evidence that extrinsic risk impacts fertility has been shown across a range of human societies (i.e. subsistence type, development stage) [33–35], although it appears that the trend towards increasing *K*-strategies is reversed at the highest levels of development [28,36]. Reversion to higher fertility norms seems to be partially explained by increasing levels of gender equality [37], emphasizing that holistic accounts of fertility will require economic, political and cultural considerations [38].

Phenotypic effects of risk should be apparent in a suite of psychological and cultural traits that motivate behaviour towards investment in *r-* or *K-* strategies [39–41]. Time orientation, one key consideration, is a psychological mechanism that may be adaptively calibrated to environmental risk [39,41]. Time orientation reflects the degree to which focus on the past, present or future guides attitudes and behaviours [42]. Present oriented individuals and cultures tend to focus on the current environment, seek out immediate pay-offs and are more likely to exhibit risky attitudes and behaviours [39,43–47]. Future orientation allows one to ‘transcend’ stimuli in the present and delay gratification, particularly towards fulfilment of future goals (e.g. education) [47–49]. Given these relationships, we predict present orientation will be associated with high extrinsic risk environments and investment in *r*-strategies while future orientation will be associated with low risk and investment in *K*-strategies.

### 2.2. Life-history theory and climate change

*K*- and *r-*strategies can impact emissions through different investment pathways. *K*-strategies produce larger energy investments *per capita*, both within parents and offspring, and high *per capita* CO_2_ emissions nationally [4,50]. By contrast, *r-*strategies are associated with lower *per capita* carbon emissions, but greater offspring quantity, increasing an individual's ‘carbon legacy’, defined as the expected future emissions produced by a parent and their surviving offspring. Carbon legacies can have a much larger impact on emissions relative to current investment changes (e.g. buying a hybrid car) [5]. Countries associated with *K*-strategies (i.e. most high-income countries) have substantially larger carbon legacies than those countries exhibiting *r* strategies (i.e. many low-income countries) [51]. A woman in Bangladesh, for example, would need to have 74 children before her carbon legacy equalled that of an American woman with one child (calculated from data in [5]). In LHT, these emission disparities can be conceived as the historical products of low risk environments that allow future benefits of increased embodied and parental investments to accrue [9]. In particular, the advances in healthcare and sanitation associated with industrialization lower mortality rates [52–54]. Lower mortality rates make it more likely that the increased levels of parental investment associated with other correlates of industrialization, such as access to education, will ultimately result in fitness benefits. It is this positive feedback between industrialization and decreased mortality that favours *K*-strategies and the inverse relationship between population growth and carbon emissions. Here, we demonstrate that national CO_2_ emissions are positively correlated with components of *K-*strategies, including increased parental investment levels and future orientation.

### 2.3. Life-history theory: resolving a paradox?

Parental investment considerations complicate solutions for anthropogenic climate change. Efforts to decrease extrinsic risk in low-income countries, while reducing demographic-linked emissions, would likely be offset by subsequent *per capita* parental investment increases with substantial carbon by-products. To reduce emissions, embodied and parental effort must be channelled towards more ‘carbon-friendly’ energy investment patterns. We question whether the more future-oriented psychologies emerging from low risk environments would allow for attention to long-term environmental catastrophe as a salient cue for parental investment. We view future orientation as an adaptive psychological response that emerges with a suite of *K*-strategies associated with industrialization/development. We wish to examine the possibility that psychological processes can be co-opted to promote more efficient energy use, providing insight for carbon emissions. Future orientation, when paired with investment in higher education, might promote behaviours that attenuate the relationship between *per capita* somatic and parental investment and carbon emissions. Future orientation might be co-opted to encourage investment in education designed to tune perception to long-term environmental impacts.

Future orientation could attenuate the relationship between investment and emissions both directly, through its promotion of pro-environmental attitudes and behaviours ([55–58], but see [59]), and indirectly, through promoting educational investment [49]. Education level, given its links to income and thus consumption, is generally positively associated with emissions [60–63]. However, this association may become non-significant, and even negative, when examined at higher levels of education, income and emissions [60,63–66]. Additionally, higher levels of education are associated with decreased pollution per dollar spent [67]. These results are consistent with studies showing that pro-environmental attitudes are related to education, but often limited to higher post-secondary levels ([68–70], but see [71]). At a national level, increases in educational attainment indirectly decrease carbon emissions by resulting in more employment in service sector jobs relative to manufacturing [72].

## 3. Material and methods

Multilevel analysis of 66 981 females from 75 countries and linear regression analysis of national-level data from the same 75 countries were used. Total fertility rates (TFRs) were used as proxies of *K-* and *r*-strategies. We restrict our analysis to those with largely completed fertility (45 years and older). Extrinsic risk was represented by the average life expectancy at birth (LEB) in the 10 years after a person was born. While LEB is a national average, we include it as an individual attribute given we use a person's age to determine LEB. Education level (EDU) was coded none, primary, secondary and post-secondary. Future orientation was a country-level indicator and represented by Hoftstede's [73] Long-Term Short-term Orientation. Long-Term Orientation is the ‘fostering of virtues oriented towards future rewards’, while ‘Short-Term Orientation stands for the fostering of virtues related to the past and present [73]’. The scale ranges from 0 (most present) to 100 (most future) and so we refer to it as future orientation (FO). The measure has been a source of some debate (see [74,75]) but scores have been validated using World, African and European Values Surveys [76,77].

Country-level effects of FO were assessed through specifying an *I* = *PAT* equation, which treats environmental impact (*I*) as a product of population (*P*), affluence (*A*) and technology (*T*) [78,79]. Carbon emissions *per capita* is used to represent *I*. Total population is used to represent *P*. GDP *per capita* is used to represent *A*. *T* is commonly represented by entering two variables into models, the percentage of GDP produced from the manufacturing sector and percentage of GDP produced from the service sector. Percentage enrolment in post-secondary education was used to represent investment in higher education. Variables were log-transformed prior to analysis, consistent with other *I* = *PAT* specifications [2]. See the electronic supplementary material for model justification and specification procedures, list of included countries and databases, orientation scores, summary statistics and correlation tables.

## 4. Results

**Figure 1.**
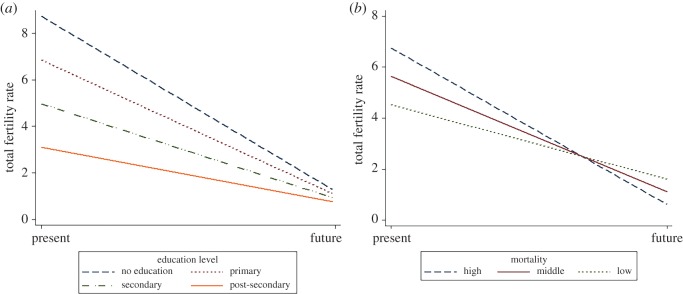
Life-history interactions with future orientation. (*a*) Effect of orientation on total fertility as a function of education level. (*b*) Effect of future orientation on total fertility as a function of mortality level.

TFR was 3.126 births when LEB and EDU were at their within-country mean and FO was at its between-country mean (table 1). Controlling for the other predictors, a 1-year increase in LEB was associated with a 0.0075 birth decrease, and a one-unit increase in EDU (e.g. none to primary) was associated with a 0.637 birth decrease. A one-unit increase in future orientation was associated with a 0.032 birth decrease. Cross-level interactions between FO and EDU and FO and LEB were significant and are graphed in figure 1*a*,*b*. There was a significant and negative covariance between the random slope of TFR on EDU and random intercept variances (*τ*_03_ = −0.249) meaning that in countries with higher TFRs (i.e. higher intercepts), the slope of the relationship between EDU and TFR is smaller and the relationship between EDU and TFR weaker.

**Table 1.** Relationship between total fertility rates, future orientation, mortality and parental investment. *N* = 66 981. EDU, education; LEB, life expectancy at birth; FO, future orientation; RI, random intercept. Parameter estimates reflect full information from maximum-likelihood robust estimation [80].

		total fertility
fixed effects	education (*γ*_10_)^a^	−0.637**
	life expectancy (*γ*_20_)^a^	−0.075**
	future orientation (*γ*_01_)^b^	−0.032**
	EDU × FO (*γ*_11_)	0.009**
	LEB × FO (*γ*_21_)	0.002**
	intercept (*γ*_00_)	3.126**
random effects	residual (*σ*^2^)	4.612**
	intercept (*τ*_00_)	2.135**
	slope (*τ*_10_) EDU	0.144**
	slope (*τ*_20_) LEB	0.015**
	covariance (*τ*_01_) LEB:EDU	0.009
	covariance (*τ*_02_) LEB:RI	−0.040
	covariance (*τ*_03_) EDU:RI	−0.191**
model summary	deviance statistics	−97 998

^a^Variable was group mean centred.

^b^Variable was grand-mean centred.

**p < 0.01.

**Figure 2.**
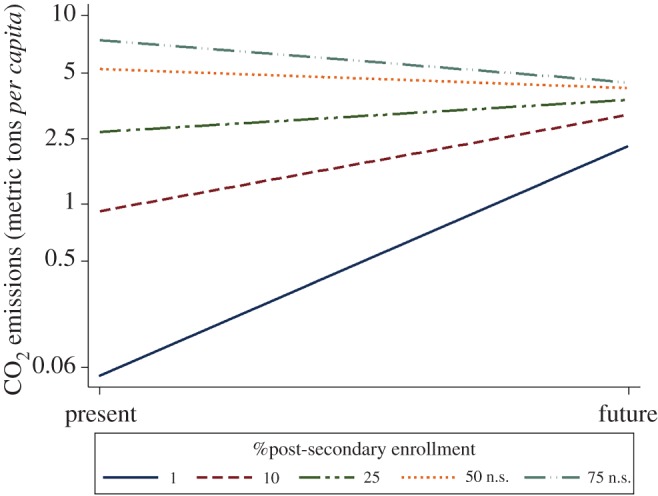
Effect of time orientation on carbon emissions *per capita* as a function of investment in education. n.s., non-significant.

Holding other contributors constant, every 1% increase in future orientation was associated with a 0.373% increase in carbon emissions *per capita* while a 1% increase in EDU was associated with a 0.527% increase in emissions (table 2). A significant interaction was detected between EDU and FO, graphed in figure 2. GDP was significantly related to emissions with a 1% increase associated with a 0.562% increase. A 1% increase in per cent of GDP from the service sector was associated with a 0.947% emissions decrease. Population and GDP from manufacturing were not significantly related to emissions.

**Table 2.** Effect of future orientation and education on carbon emissions. All variables were log-transformed. *N* = 75. FO, future orientation.

	CO_2_ emissions (%)
future orientation	0.373*
education	0.527**
FO × education	−0.337*
GDP *per capita*	0.562**
FO × GDP	−0.038
total population	0.058
%GDP from manufacturing	−0.059
%GDP from service	−0.947**
const.	3.624*
*R*^2^	0.89

**p < 0.01; *p < 0.05.

## 5. Discussion

LHT provides insight into the demographic and consumption dynamics driving global carbon emissions. Life-history components of *K-*strategies, including low mortality, high investment and future orientation, are negatively correlated with TFRs across 67 000 women and 75 countries. Furthermore, future orientation appears to significantly interact with risk and investment regimes. Differences in TFRs across education levels *diminished* as countries became more future oriented, suggesting that education, at any level, was associated with longer delays in reproduction and higher embodied and parental investment within future-oriented countries (figure 1*a*). As future orientation increased, the greater rate of decline in predicted TFRs at high risk levels (figure 1*b*) resulted in a crossover interaction where high risk environments actually predicted lower fertility than low risk environments. This result, seemingly inconsistent with LHT, demonstrates the importance of including time orientation in investigations of how human LHs respond to local risk environments.

Future orientation was positively associated with carbon emissions in 75 countries after controlling for the contributions of population, affluence and technology (table 2). Yet future orientation also interacted with education to attenuate the association between investment and emissions. As enrolment in post-secondary education increased, the association between future orientation on CO_2_ emissions decreased and did not significantly impact emissions in countries with the highest enrolment levels (figure 2), suggesting that education may help mitigate the increased CO_2_ emissions promoted by *K-*strategies.

Emissions from high-income countries will continue to comprise the majority of global carbon output and so it is these countries who are, and will be, responsible for increases in global risk levels. Yet, it is low-income countries who are, and will be, most impacted by these risks (e.g. extreme weather, civil unrest) [3,81]. These increasing levels of extrinsic risk combined with the tendency for low-income countries to exhibit *r*-strategies (including less future orientations) point to a catastrophic possibility—rapid population growth within those countries where growth has the largest impact on carbon emissions. To short-circuit feedback loops between population growth in low-income countries and unsustainable investment in high-income countries, future research should determine how future orientation and education can be leveraged to slow the global march towards earth's carrying capacity. Promising areas include studies providing evidence that future orientations can be ‘taught’ within the classroom [82]. Future studies should also include attributes correlated with education, including wealth, to better isolate the relationships between education and time orientation (and vice versa).

## 6. Conclusion

LHT represents an evolutionary framework for explaining the demographic and consumption patterns driving anthropogenic climate change. Patterns of global carbon emissions are the partial product of human life histories, themselves a response to local selection environments. LHT helps us to identify forces, both external (e.g. risk) and internal (e.g. psychology), that interact within local environments to shape reproduction strategies. Thus, LHT can provide an account of climate change grounded in human evolution. Our current results suggest that while both *r-* and *K*-selected strategies can lead to higher carbon emissions, future orientation may mitigate expected carbon emissions through lowering fertility and interacting with parental investment, particularly in the context of higher education. Nevertheless, evidence for fertility rate increases in countries with the highest levels of development [36] suggests more research is needed into how orientation, and evolutionary approaches in general, can explain global patterns of human LH [28]. Future orientation and related psychological processes (e.g. delay discounting, impulsivity) may present points of intervention to alter human behaviours fuelling anthropogenic climate change.

## Supplementary Material

File 1: Variables Definitions and Model Specifications- This supplementary file contains definitions of variables included in the model, more information and model specifications, and variance accounted for by model 1

## Supplementary Material

File 2: Supporting Data used in Model 1 and Model 2- This supplementary file includes all data used in the analyses. Variable names are prefixed by the specific model they were included in (e.g., "model 1)

## Data accessibility

The datasets supporting this article have been uploaded as part of the electronic supplementary material.
