# Supplementary material for: Life-history theory and climate change: resolving population and parental investment paradoxes
Source: R Soc Open Sci. 2016 Nov 30;3(11):160470. doi: 10.1098/rsos.160470 (PMC5180129; doi:10.1098/rsos.160470)
Supplement: File 1: Variables Definitions and Model Specifications- This supplementary file contains definitions of variables included in the model, more information and model specifications, and variance accounted for by model 1 [file rsos160470supp1.pdf]

Caudell_Variable Definitions_Model Specifications_ESM

Model 1: Variables

Data were collected from the World Values Survey (http://www.worldvaluessurvey.org/), European Values Survey (http://www.europeanvaluesstudy.eu/), and surveys available at the Integrated Public Use Microdata Series-International from the Minnesota Population Center, including the Demographic Health Surveys (http://www.ipums.org/)(1–3). As stated in the Methods action, education level (EDU) was coded into four categories, “none”, “primary”, “secondary”, and “post-secondary”. Surveys that did not already use this coding system but instead recorded an individual’s education in years were recoded into one of these four categories using the following criteria; 0-2 years = “none”, 3-5 years = “primary”, 6-10 years = “secondary”, 10+ years = “post-secondary”. Life expectancy at birth (LEB) is defined as the number of years of life remaining at birth. Data on average life expectancy at birth in the 10 years after an individual's birth was calculated from data collected at the World Bank (http://data.worldbank.org/) (4). Education level and LEB were group-mean centered (national education level and average LEB 10 years after birth) the suggested centering technique to ensure correct interpretation of the level-1 effects (5)(http://data.worldbank.org/). For Hofstede’s time orientation measure 72 of the 75 countries had country-specific future orientation scores while three countries were assigned regional scores, including Ethiopia and Kenya to the East Africa region and Sierra Leone to the West African region . As a level-2 effect future orientation was grand mean centered using the global mean. Future orientation scores, database from which the fertility and education data were taken and associated years of collection are provided in Table S2. Table S3 provides pairwise correlations for model 1 variables.

Table S1| Descriptive statistics for Model 1. N=66,981. Std. Dev. = standard deviation.

Variable	mean	std. dev.	min	max
Total fertility rate (TFR)	3.60	2.75	0.00	20.00
Education level (EDU)	1.19	0.97	0.00	3.00
Future orientation (FO)	45.54	24.88	0.00	100.00
Life expectancy at birth (LEB)	63.32	10.23	27.08	80.42

Table S2| Data sources, years, and future orientation scores. WVS is the World Values Survey. EVS is the European Values Survey. IPUMS is Integrated Public Use Microdata Series-International from the Minnesota Population Center.

Country	Year(s)	Database	FO
Albania	2008	IPUMS	61
Algeria	2002	WVS	26
Argentina	2001	IPUMS	20
Armenia	2005	IPUMS	61
Australia	2005	WVS	21
Austria	2001	EVS	60
Azerbaijan	2006	IPUMS	61
Bangladesh	2007	IPUMS	47
Belarus	1999	WVS	81
Belgium	1999	EVS	82
Bosnia and Herzegovina	1998	WVS	70
Brazil	2000	WVS	44
Bulgaria	1999	WVS	69
Burkina Faso	2003	IPUMS	27
Canada	2000	WVS	36
Chile	2002	WVS	31
China	1990	IPUMS	87
Colombia	2005	IPUMS	13
Croatia	1999	EVS	58
Czech Republic	1999	WVS	70
Denmark	1999	EVS	35
Dominican Republic	2007	IPUMS	13
Egypt	2005	IPUMS	7
El Salvador	1999	WVS	20
Estonia	1996	WVS	82
Ethiopia	1997	WVS	32
Finland	1996	WVS	38
France	2006	WVS	63
Georgia	2008	WVS	38
Germany	2006	WVS	83
Ghana	2003	WVS	4
Greece	2001	EVS	45
Hungary	2001	IPUMS	58
Iceland	1999	EVS	28
India	2006	IPUMS	51
Indonesia	2007	IPUMS	62

Iran (Islamic Republic of)	2006	WVS	14
Iraq	1997	WVS	25
Ireland	1999	EVS	24
Israel	1995	WVS	38
Italy	2005	WVS	61
Japan	2005	WVS	88
Jordan	2002	WVS	16
Kenya	2009	IPUMS	32
Kyrgyzstan	1999	IPUMS	66
Latvia	1996	WVS	69
Lithuania	1997	WVS	82
Luxembourg	1999	EVS	64
Malaysia	2006	WVS	41
Mali	1998	IPUMS	20
Malta	1999	EVS	47
Mexico	1996	WVS	24
Morocco	2003	IPUMS	14
Netherlands	2006	WVS	67
New Zealand	2004	WVS	33
Nigeria	2008	IPUMS	13
Pakistan	2007	IPUMS	50
Peru	2000	IPUMS	25
Philippines	2003	IPUMS	27
Poland	2005	WVS	38
Portugal	1999	EVS	28
Puerto Rico	1990	IPUMS	0
Republic of Korea	1990	WVS	100
Republic of Moldova	2005	WVS	71
Romania	2002	WVS	52
Russian Federation	1999	WVS	81
Rwanda	2005	IPUMS	18
Saudi Arabia	2003	WVS	36
Serbia	2006	WVS	52
Sierra Leone	2008	IPUMS	9
Slovakia	1999	EVS	77
Slovenia	2002	IPUMS	49
South Africa	1998	IPUMS	34
Spain	2007	WVS	48
Sweden	1999	EVS	53
Switzerland	2000	EVS	74
Taiwan	2006	WVS	93
Turkey	2003	IPUMS	46
Uganda	2000	IPUMS	24
Ukraine	2007	IPUMS	86
United Kingdom	1998	WVS	51
United Republic of Tanzania	2004	IPUMS	34
United States of America	1999	WVS	26
Venezuela	2001	IPUMS	16
Vietnam	2002	IPUMS	57
Zambia	2007	IPUMS	30

Zimbabwe	2005	IPUMS	15

Table S3| Variable correlations for Model 1. N=66,981.

Variable	TFR	EDU	FO	LEB
TFR	1			
Edu	-0.48*	1		
FO	-0.38*	0.35*	1	
LEB	-0.38*	0.53	0.42	1

$^{**}p<.01$. $^{*}p<.05$

Model 2: Variables

Model 2 variables were taken from the World Bank (http://data.worldbank.org/) (Table S4). Variables were chosen as they are commonly used in I=PAT models (see 6 for review). GDP per capita is gross domestic product in current US dollars divided by midyear population. Total Populations are midyear estimates. Percentage enrollment in post-secondary education (also referred to as tertiary education) represents total enrollment regardless of age. Percentage GDP from manufacture is value added, meaning it is "the net output of a sector after adding up all outputs and subtracting intermediate inputs. It is calculated without making deductions for depreciation of fabricated assets or depletion and degradation of natural resources". "Carbon emissions per capita are expressed as metric tons and are emissions from the burning of fossil fuels and the manufacture of cement and include CO_2 produced during consumption of solid, liquid and gas fuel and gas flaring"(7). Given log transformation prior to analysis, coefficients are interpreted as percentage increases in carbon emissions per capita given a 1% increase in the independent variable. With the exception of future orientation scores all variables for Model 2 were collected in 2000.

Table S4| Descriptive statistics for Model 2. N=75. Std. Dev. = standard deviation.

**Variable**	**mean**	**std. dev.**	**min**	**Max**
C02 Emissions per Capita	5.240	4.613	0.065	20.208
Future Orientation	46.434	23.680	7.000	100.000
Post-Sec. Education	33.531	21.451	0.676	82.439
GDP per Capita	12880.650	16526.710	136.630	73270.630
Total Population	66300000	188000000	281205	1260000000
% GDP from Manufacture	17.619	6.863	3.549	40.021
% GDP from Service	56.560	12.996	13.250	80.982

Table S5| Variable Correlations for Model 2. CO_2 = CO_2 emissions per capita. FO = Future orientation. EDU= Percentage enrolled in post-secondary education. GDP=GDP per capita. Pop= Total population. GDP_Man = Percentage GDP from manufacture sector. GDP_Serv = Percentage GDP from service sector. N=75.

**Variable**	**CO_2**	**FO**	**EDU**	**GDP**	**Pop**	**GDP_Man**	**GDP _Serv**
CO_2	-						
FO	0.242*	-					
EDU	0.631**	0.333*	-				
GDP	0.740**	0.182	0.538**	-			
Population	-0.084	0.161	-0.196	-0.122	-		
GDP_Man	0.171	0.361*	0.361**	0.084	0.266*	-	
GDP_Serv	0.561**	0.194	0.583**	0.644**	-0.160	0.1445	-

** p<.01. * p<.05

Model 1 Justification

Across the 75 countries in Model 1, the number of individuals ranged from 177 to 1000 per country, with an average cluster size of 351.5 individuals. Model 1 was tested using a multilevel modeling approach. This approach was appropriate given fertility behavior within the same country, due to shared sociocultural, biological, and physical environments, is more likely to be correlated than fertility behaviors between countries and thus observations lack independence. Multilevel models compensate for the likelihood of non-independent observations by partitioning variance into variance accounted for by within and between group levels.

Through this partitioning, multilevel models also avoid committing the ecological fallacy, which can occur when inferences about behaviors of individuals are based solely upon aggregate measures of the group to which those individuals belong (8). This is potentially a serious problem in the cross-cultural studies of life history as the majority of studies (9–11), rely on aggregate measures.

Model 1 Specification

A random intercept model (sometimes referred to as “baseline”, “null” or “unconditional” model) was first specified to justify the use of a multilevel modeling approach through calculation of the intra-class correlation (ICC). This model partitioned TFR into variance existing at the within- and between-country levels. The ICC was .38 indicating that about 38% of the variance in individual TFRs is associated with countries (i.e., between group) while around 62 % is associated with individuals (i.e., within-group). Alternatively, the ICC can be interpreted as the extent to which individuals within the same country are more alike compared with individuals of a different country (5). Specifically, the TFRs within the same country are 38% more similar relative to TFRs within a different country and so are not independent observations.

Model specification proceeded by adding level-one predictors (i.e., EDU, LEB) as fixed effects (e.g., the regression coefficient of TFR on education could not vary across countries) and then as random effects (i.e., the regression coefficient of TFR on education could vary across countries). Random effects are necessary as the effect of education and LEB on TFR (i.e., regression coefficient) is hypothesized to vary across countries, in part, due to differences in a countries future orientation. The level-two predictor of future orientation was then regressed on the random slope as predicted by the regression of TFR on EDU and LEB. Regression of FO on the random slopes allowed examination of whether FO had a significant effect on the

relationship between EDU and TFR and LEB and TFR. Finally, cross-level interaction effects were specified between FO and EDU and LEB (see discussion in Manuscript). Likelihood-ratio tests were used to determine whether a model provided a significantly better fit (i.e., $p<.05$) compared to the previous model (12). All models provided a significantly better fit compared to the previously specified model. Given data was collected in different years, year was entered as a control variable but did not have a significant impact on TFR, after controlling for EDU, LEB, and FO. Additionally, earlier models included the predictor of Gross National Income per capita (GNI per capita) given high correlations between education and income. As a county level effect, GNI per capita did not have a significant effect on the relationship between LEB and TFR or EDU and TFR. We suspect that if we have included income at the individual level then results may have differed, although the inclusion of wealth was not permitted given it was gathered differently across the several surveys used.

M*plus* v6.1 and Stata v13 were used for analysis. Analysis of the same model in two programs holds the benefit of cross-checking model specification and estimation. Stata v13 uses a maximum likelihood estimator while the default estimator in *Mplus 6*.1 is a maximum likelihood robust estimator. Results from both statistical packages were almost identical providing strong evidence of proper model specification and estimation.

Calculation of variance accounted for by the final model is complicated as variance exists both within and between countries. For equations 1-3 below, *a* = residual variance associated with unconditional/null model. *b*= residual variance associated with final model c = residual variance from random intercept random slope model without level 2 predictors**.**

Equation 1: Residual variance accounted for by all covariates at both levels

$$R^2 = \frac{(\sigma^2 + \tau 00^2)a - (\sigma^2 + \tau 00^2)b}{(\sigma^2 + \tau 00^2)a} = .272$$

Equation 2: Residual variance accounted for within countries by all covariates

$$R^2 = \frac{(\sigma^2)a - (\sigma^2)b}{(\sigma^2)a} = .137$$

Equation 3: Residual variance accounted for between countries by all covariates at between levels

$$R^2 = \frac{(\tau 00^2)a - (\tau 00^2)b}{(\tau 00^2)a} = .598$$
